# A Selective FGFR inhibitor AZD4547 suppresses RANKL/M-CSF/OPG-dependent ostoclastogenesis and breast cancer growth in the metastatic bone microenvironment

**DOI:** 10.1038/s41598-019-45278-w

**Published:** 2019-06-19

**Authors:** Jinho Kang, Yoon Ji Choi, Bo Yeon Seo, Ukhyun Jo, Serk In Park, Yeul Hong Kim, Kyong Hwa Park

**Affiliations:** 1 The BK21 Plus Program, Korea University College of Medicine, Seongbuk-Gu, Seoul Republic of Korea; 2 Division of Oncology/Hematology, Department of Internal Medicine, Korea University College of Medicine, Seongbuk-Gu, Seoul Republic of Korea; 3 Department of Biochemistry and Molecular Biology, Korea University College of Medicine, Seongbuk-Gu, Seoul Republic of Korea; 4 Department of Medicine, Vanderbilt University School of Medicine, Nashville, TN USA

**Keywords:** Breast cancer, Cancer microenvironment, Cancer prevention, Targeted therapies

## Abstract

Aberrant activation of fibroblast growth factor receptor (FGFR) signalling contributes to progression and metastasis of many types of cancers including breast cancer. Accordingly, FGFR targeted tyrosine kinase inhibitors (TKIs) are currently under development. However, the efficacy of FGFR TKIs in the bone microenvironment where breast cancer cells most frequently metastasize and also where FGFR is biologically active, has not been clearly investigated. We investigated the FGFR-mediated interactions among cancer and the bone microenvironment stromal cells (osteoblasts and osteoclasts), and also the effects of FGFR inhibition in bone metastasis. We showed that addition of culture supernatant from the MDA-MB-134-VI *FGFR*-amplified breast cancer cells-activated FGFR siganalling in osteoblasts, including increased expression of RANKL, M-CSF, and osteoprotegerin (OPG). Further *in vitro* analyses showed that AZD4547, an FGFR TKI currently in clinical trials for breast cancer, decreased RANKL and M-CSF, and subsequently RANKL and M-CSF-dependent osteoclastogenesis of murine bone marrow monocytes. Moreover, AZD4547 suppressed osteoclastogenesis and tumor-induced osteolysis in an orthotopic breast cancer bone metastasis mouse model using FGFR non-amplified MDA-MB-231 cells. Collectively, our results support that FGFR inhibitors inhibit the bone microenvironment stromal cells including osteoblasts and osteoclasts, and effectively suppress both tumor and stromal compartments of bone metastasis.

## Introduction

Previous genomic profiling studies demonstrated that several potential target pathways are aberrantly regulated in breast cancer, including the fibroblast growth factors (FGF)/FGF receptor (FGFR) signalling pathway^1^. The FGFR family comprises 4 main members (i.e. FGFR1– 4), some of which have multiple isoforms because of alternative splicing^2^. FGFRs are expressed in various cell types and regulate key cellular behaviours. It is widely known that FGFRs play major roles in tumour cell proliferation, differentiation, survival, and migration as well as angiogenesis^3,4^. FGFR is one of the first factors amplified in breast cancer, and shows increased levels in 8–15% of breast cancers. *FGFR* gene amplification has been shown to be associated with early relapse and poor survival in breast cancer^5,6^. Additionally, FGFRs function to regulate bone formation at all stages of the osteogenic lineage through 2 different mechanisms: (1) indirect stimulation of osteoblasts through induction of receptor activator of nuclear kappa B ligand (RANKL) and (2) direct inhibition of osteoclast precursors by counteracting macrophage colony-stimulating factor (M-CSF) signalling^7,8^.

Bone metastasis is a common and often incurable complication in many types of cancer. Interactions among cancer cells, osteoblasts, osteoclasts, and the bone matrix are essential for bone metastases^9^. FGFR is a key regulator of bone metastases and consequential morbidity in FGFR-amplified breast cancer through its biological interactions in the bone microenvironment. Although FGFR amplification in breast cancer has not been shown to be directly associated with bone metastases, the prognosis of patients with FGFR-driven tumours is poor. Furthermore, the interactions among FGFR-amplified breast cancer cells and other cells and factors in the bone microenvironment, including osteoblasts, osteoclasts, and the bone matrix, have not been thoroughly investigated.

Because of their prominent roles in various cancers, FGFRs have become important targets for drug development^10^. These efforts have led to the discovery of several FGFR inhibitors, including dovitinib, BGJ398, ponatinib, and LY2874455, all of which have entered clinical trials as potential anticancer drugs. For example, dovitinib showed antitumour activity against various cancer types with FGFR amplification and functions by altering the microenvironment through inhibition of stromal cells and through its direct cytotoxicity towards cancer cells^11–15^. However, the clinical development of dovitinib for most cancer types was discontinued by the developer.

AZD4547 is an orally bioavailable, highly selective, potent, ATP-competitive small molecule inhibitor of FGFR1, 2, and 3. AZD4547 selectively inhibits FGFR phosphorylation and represses the proliferation of cancer cells by inhibiting FGFR signalling^16,17^. Recently, it was reported that AZD4547 treatment inhibited the growth of various cancer types with FGFR amplification, including breast cancer^18^. Based on its potency and selectivity, AZD4547 is a promising agent for patients with FGFR-amplified breast cancer, for which an effective pharmacodynamics marker should be developed.

In the present study, we demonstrated that AZD4547 suppressed the mRNA and protein expression of RANKL, M-CSF, and osteoprotegerin (OPG) in osteoblasts induced with an MDA-MB-134-VI cells supernatant. We confirmed that AZD4547 inhibited osteoclastogenesis in mouse bone marrow monocytes (BMMs) induced by a combination of M-CSF and RANKL. We also investigated the effects of AZD4547 in the bone microenvironment using FGFR-resistant breast cancer cells *in vivo*.

## Results

### FGFR-expressing breast cancer cells are sensitive to AZD4547

**Figure 1:**
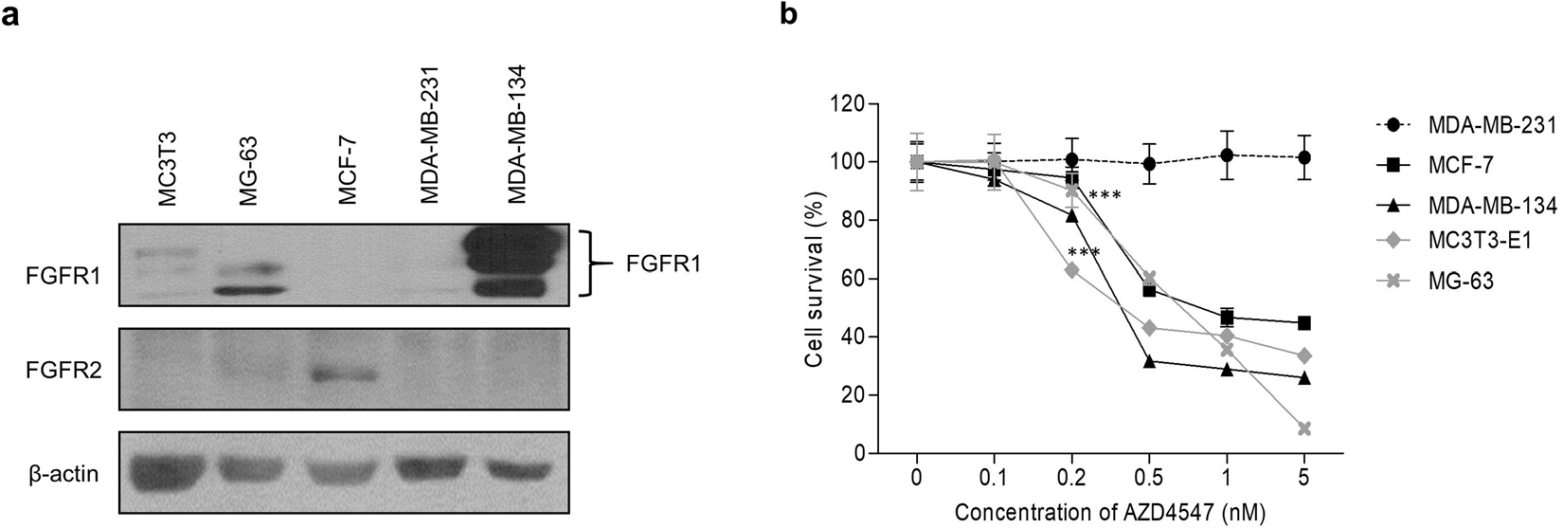
Evaluation of AZD4547 sensitivity of breast cancer and pre-osteoblast cells. (**a**) Expression levels of fibroblast endothelial growth factor receptor 1 (FGFR1) and FGFR2 in 3 breast cancer cell lines and osteoblastic cells were evaluated by western blotting. β-Actin was used as a loading control. (**b**) Changes in cell survival following treatment with AZD4547 were determined in MTT assays. The percentage of viable cells after treatment with the indicated concentrations of AZD4547 for 24 h is shown. Each data point represents the mean of 6 independent determinations with the standard deviation. ***p < 0.001 compared to MDA-MB-231 cells in two-way ANOVA.

Two luminal-type breast cancer cell lines, MDA-MB-134-VI and MCF-7, were selected based on their FGFR expression levels. MDA-MB-231 cells were included as a negative control. The murine pre-osteoblastic cell line (MC3T3-E1 Subclone 4) and human osteosarcoma cell line (MG-63) were also evaluated for FGFR expression. As shown in Fig. 1a, FGFR1 expression levels were highest in FGFR-amplified MDA-MB-134-VI cells, and FGFR2 expression was detected in MCF-7 cells. In contrast, no FGFR expression was detected in MDA-MB-231 cells. FGFR1 protein expression was also detected in MC3T3-E1 Subclone 4 osteoblastic cells and MG-63 human osteosarcoma cells. Next, to determine whether AZD4547 sensitivity depends on the FGFR amplification status, a cell viability assay was performed after AZD4547 treatment. As shown in Fig. 1b, the percentage of viable FGFR-expressing cells decreased significantly after AZD4547 treatment (0.5 nM) in a dose-dependent manner (with 60%, 56%, 43%, and 32% survival of MG-63, MCF-7, MC3T3-E1, and MDA-MB-134-VI cells, respectively, *p* < 0.05). In contrast, the FGFR non-amplified cell line (MDA-MB-231) was not sensitive to any concentration of AZD4547. Additionally, 2 osteoblast cell lines showed similar dose-dependent sensitivities to AZD4547. These results indicate that AZD4547 inhibited the growth of osteoblastic cells and FGFR-amplified/overexpressing breast cancer cells.

### Osteoblastic FGFR is activated by the supernatant of MDA-MB-134-VI cells

**Figure 2:**
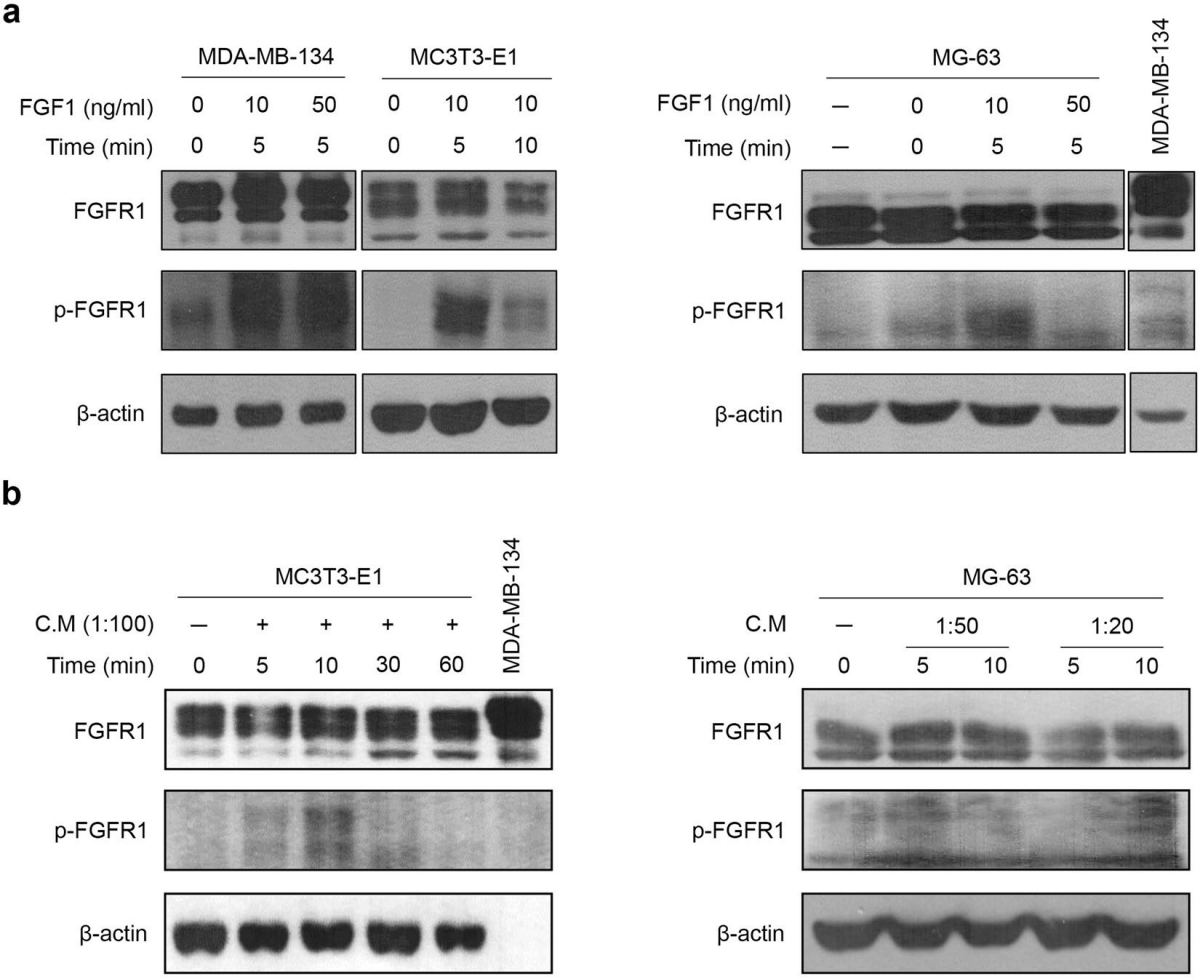
Evaluation of FGFR activation in pre-osteoblast cells induced by MDA-MB-134-VI cell supernatant. The changes in FGFR phosphorylation levels after stimulation with FGF1 (**a**) and MDA-MB-134-VI supernatants (**b**) were investigated by western blot analysis. The cells were pre-stimulated with FGF1 and MDA-MB-134-VI supernatants under serum-free conditions for 24 h and incubated for the indicated times in the presence or absence of FGF1 and MDA-MB-134-VI supernatants. β-Actin was used as a loading control. C.M, MDA-MB-134-VI supernatant.

Previous studies showed that the FGF/FGFR signalling pathway plays a critical role in regulating osteoclastogenesis and the proliferation of FGFR-amplified breast cancer cells. To investigate whether FGFR in osteoblasts residing in the bone microenvironment is activated by cancer cells in a paracrine manner, the effects of culture supernatants from FGFR-amplified MDA-MB-134-VI cells on cancer cell-osteoblasts interactions were evaluated. As shown in Fig. 2a, FGF1 significantly upregulated the phosphorylation of FGFR1 in MC3T3-E1 and MG-63 cells as well as MDA-MB-134-VI cells. As the MDA-MB-134-VI breast cancer cell line is known to harbour an *FGFR* amplification, we predicted that the supernatants of MDA-MB-134-VI cells contained abundant FGF and other ligands that activate FGFR. To test this hypothesis, we added various dilutions of the MDA-MB-134-VI supernatant to cultures of MC3T3-E1 and MG-63 cells. The results, shown in Fig. 2b, confirmed that lower levels of MDA-MB-134-VI supernatants (1:100 or 1:20 dilution) increased FGFR1 phosphorylation levels in MC3T3-E1 and MG-63 cells. These results suggest that FGFR in osteoblasts in the bone microenvironment were activated by MDA-MB-134-VI cells in a paracrine manner.

### AZD4547 inhibits MDA-MB-134-VI supernatant-induced expression of RANKL/M-CSF/OPG in osteoblasts

**Figure 3:**
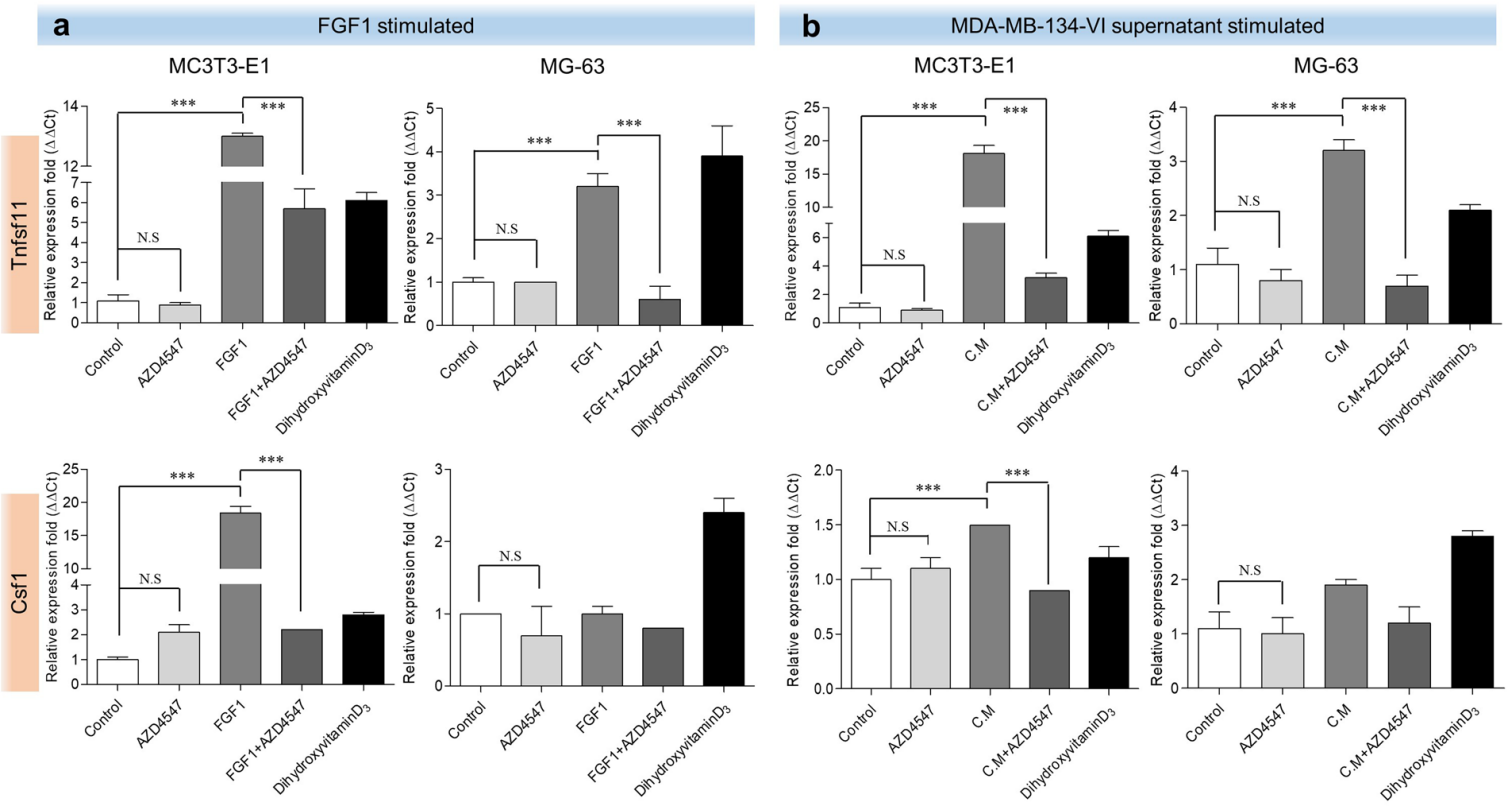
Effects of AZD4547 on expression of RANKL, M-CSF, and osteocalcin following FGFR activation induced by FGF-1 and MDA-MB-134-VI supernatant. Effects of AZD4547 on FGF1-stimulated (**a**) and MDA-MB-134-VI supernatant-stimulated (**b**) expression of osteoblast-related genes (Tnfsf11 and Csf1) in pre-osteoblast cells were examined by quantitative RT-PCR. Cells were stimulated with FGF1 (10 ng/mL) or MDA-MB-134-VI supernatant (diluted 1:20), and then treated with AZD4547 (0.2 nM) for 2 h before total RNA lysates were prepared. Each data point represents the mean value acquired from 3 experiments with the standard error. DihydroxyvitaminD_3_ is mean as positive control of osteoblasts. C.M, MDA-MB-134-VI supernatant. ***p < 0.001, in one-way ANOVA.

**Figure 4:**
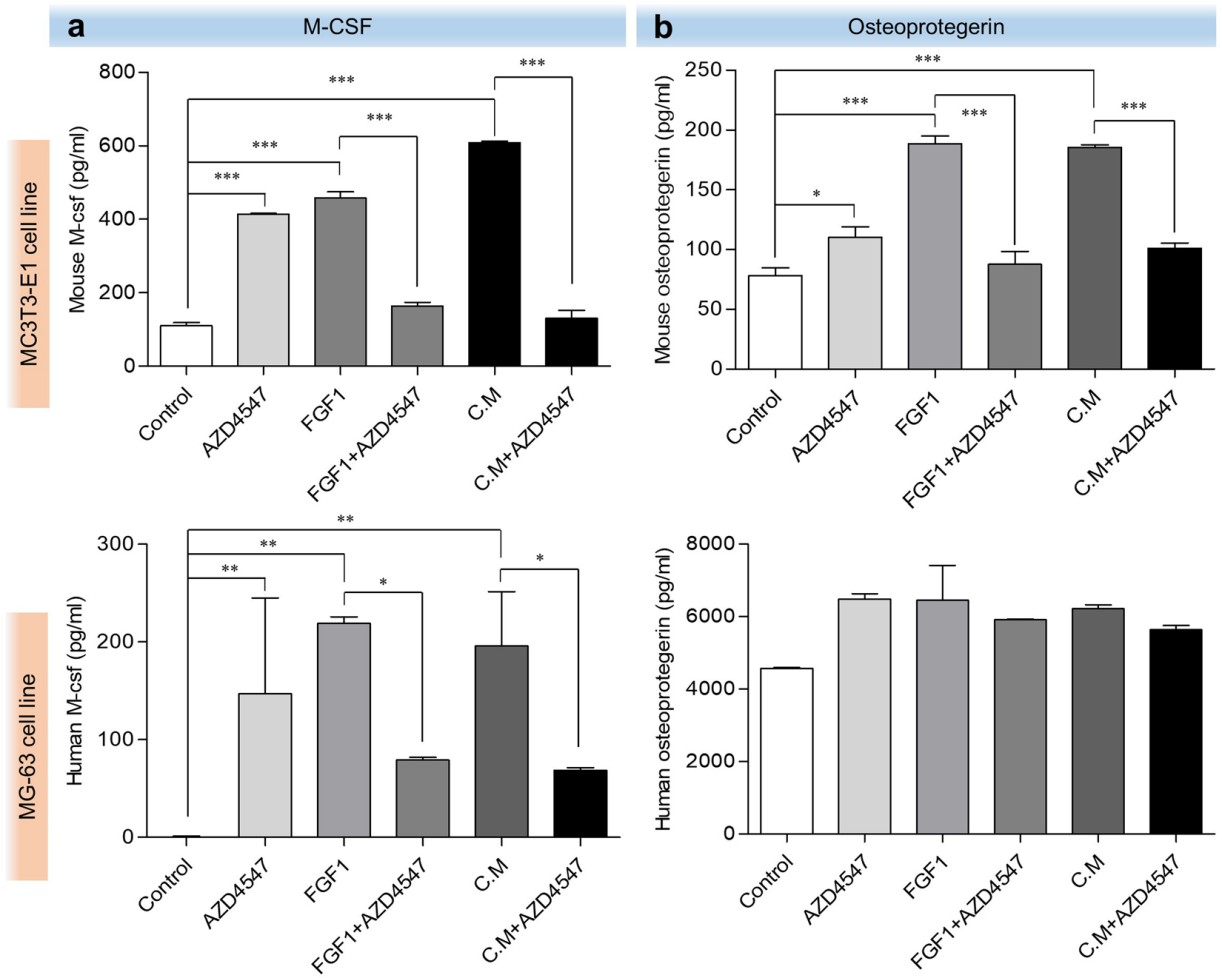
M-CSF and OPG expression levels induced by FGF-1 or MDA-MB-134-VI supernatants are reduced by AZD4547. The effects of AZD4547 on FGF1 (10 ng/mL)- or MDA-MB-134-VI supernatants (diluted 1:20)-induced M-CSF (**a**) and OPG (**b**) expression was examined using ELISA after treatment with AZD4547 (0.2 nM) in MC3T3 and MG-63 cells. The optical density of each well at 450 nm was determined using an iMARK^TM^ microplate reader. Each point represents the mean of 3 independent determinations with the standard deviation. C.M, MDA-MB-134-VI supernatant. ***p < 0.001 in one-way ANOVA.

RANKL, M-CSF, and OPG are cytokines released by osteoblasts and induce pre-osteoclasts to develop into osteoclasts. To determine whether AZD4547 can prevent FGF1-induced RANKL/M-CSF expression in osteoblasts, we examined the relative expression levels of Tnfsf11 (encoding *Rankl*) and Csf1 (encoding *M-csf*) mRNA by quantitative RT-PCR. As shown in Fig. 3a, stimulation with 10 ng/mL FGF1 significantly increased the mRNA expression of Tnfsf11 and Csf1 compared to the levels in untreated negative control MC3T3-E1 and MG-63 cells. Treatment with AZD4547 (0.2 nM) significantly decreased FGF1-induced Tnfsf11 and Csf1 expression in MC3T3-E1 cells. However, MG-63 cells were not statistically significant in expression of Csf1. Additionally, to detect cell-cell interactions between the FGFR-amplified MDA-MB-134-VI breast cancer cell line and osteoblasts, the effect of supernatants from MDA-MB-134-VI cells was assessed. Stimulation with diluted MDA-MB-134-VI cell supernatant (1:100 or 1:20) significantly increased the mRNA expression of Tnfsf11 in MC3T3-E1 and MG-63 cells via FGFR activation (Fig. 3b). Treatment with AZD4547 (0.2 nM) significantly decreased MDA-MB-134-VI supernatant-induced expression of Tnfsf11 in MC3T3-E1 cells by inhibiting FGFR activation. However, Csf1 expression was not statistically significant in MG-63 cells. Additionally, to determine whether AZD4547 can prevent FGF1- or MDA-MB-134-VI supernatant-induced M-CSF and OPG expression in osteoblasts, we examined the expression of M-CSF and OPG by enzyme-linked immunosorbent assay (ELISA). As shown in Fig. 4a,b, stimulation with 10 ng/mL FGF1 and with the diluted MDA-MB-134-VI cell supernatant significantly increased M-CSF and OPG expression compared to the levels in untreated negative control MC3T3-E1 cells supernatants. AZD4547 was significantly decreased FGF1- or MDA-MB-134-VI supernatant-induced M-CSF and OPG expression in the supernatant of MC3T3-E1 cells. However, supernatant of MG-63 cells was not statistically significant in expression of M-CSF and OPG. Another selective FGFR inhibitor BGJ398, was used to validate the effect of FGFR inhibition on osteoblasts shown by AZD4547. Both MC3T3-E1 and MG-63 cells showed a similar sensitivity to BGJ398 as AZD4547. The relative expression levels of Tnfsf11 and Csf1 mRNA by quantitative RT-PCR and expression levels of M-CSF and OPG by ELISA were performed. Consistent with AZD4547, stimulation with FGF1- or MDA-MB-134-VI cell supernatant increased the mRNA expression of Tnfsf11 and Csf1 in MC3T3-E1 and MG-63 cells. Treatment with BGJ398 decreased FGF1- or MDA-MB-134-VI supernatant-induced expression of Tnfsf11 and Csf1 by inhibiting FGFR activation in MC3T3-E1 and MG-63 cells. However, the mRNA expression of Csf1 was not statistically significantly in MC3T3-E1 and MG-63 cells (see Supplementary Fig. S2). Also, stimulation with FGF1 and MDA-MB-134-VI cell supernatant significantly increased M-CSF and OPG expression levels in MC3T3-E1 and MG-63 cells supernatants. Treatment with BGJ398 significantly decreased FGF1- or MDA-MB-134-VI supernatant-induced M-CSF and OPG expression in the supernatants of MC3T3-E1 and MG-63 cells. However, MG-63 cells were not statistically significant in OPG expression level (see Supplementary Fig. S3). These results indicate that Tnfsf11, Csf1, and OPG expression in osteoblasts were induced by MDA-MB-134-VI supernatant via FGFR activation and was inhibited by AZD4547.

**Figure 5:**
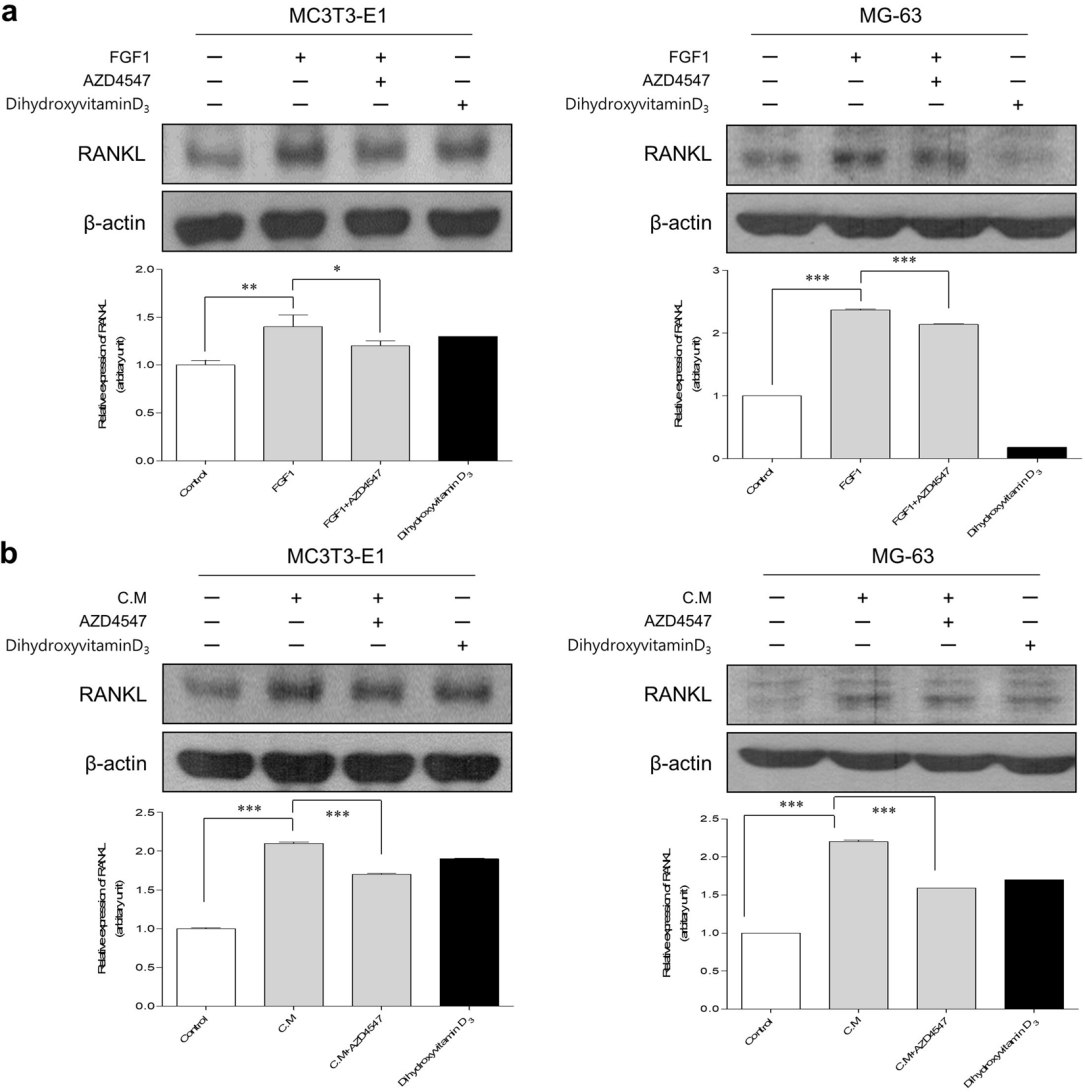
Protein expression levels of RANKL induced by FGF-1 or MDA-MB-134-VI supernatants are reduced by AZD4547. The effects of AZD4547 on FGF1 (10 ng/mL)-induced (**a**) and MDA-MB-134-VI supernatants (1:20)-induced (**b**) RANKL protein expression was examined by western blot analysis after treatment with AZD4547 (0.2 nM). Expression was evaluated by quantitative densitometry using QuantityONE v461. Each point represents the mean of 3 independent determinations with the standard error. C.M, MDA-MB-134-VI supernatant. *p < 0.05, **p < 0.01, ***p < 0.001 in one-way ANOVA.

Changes in osteoblastic RANKL protein levels in response to FGF1, MDA-MB-134-VI supernatant, or AZD4547 were assessed by western blotting. In agreement with the RT-PCR data, both FGF1 and the MDA-MB-134-VI supernatant significantly increased the protein levels of RANKL in MC3T3-E1 and MG-63 cells, and treatment with AZD4547 significantly attenuated this increase (Fig. 5a). Quantitative densitometry analysis revealed that stimulation with FGF1 and the MDA-MB-134-VI supernatant significantly increased RANKL protein levels by 1.3- and 2.2-fold, respectively, while AZD4547 significantly decreased these increases (to relative protein expression levels of 1.1- and 1.7-fold, respectively; Fig. 5a,b). These results suggest that osteoblasts were induced to produce RANKL/M-CSF/OPG by stimulation with FGF1 or the MDA-MB-134-VI supernatant, whereas AZD4547 significantly inhibited this osteoblastic activation mediated by stimuli from cancer cells.

### AZD4547 inhibits RANKL- and M-CSF-induced osteoclastogenesis

**Figure 6:**
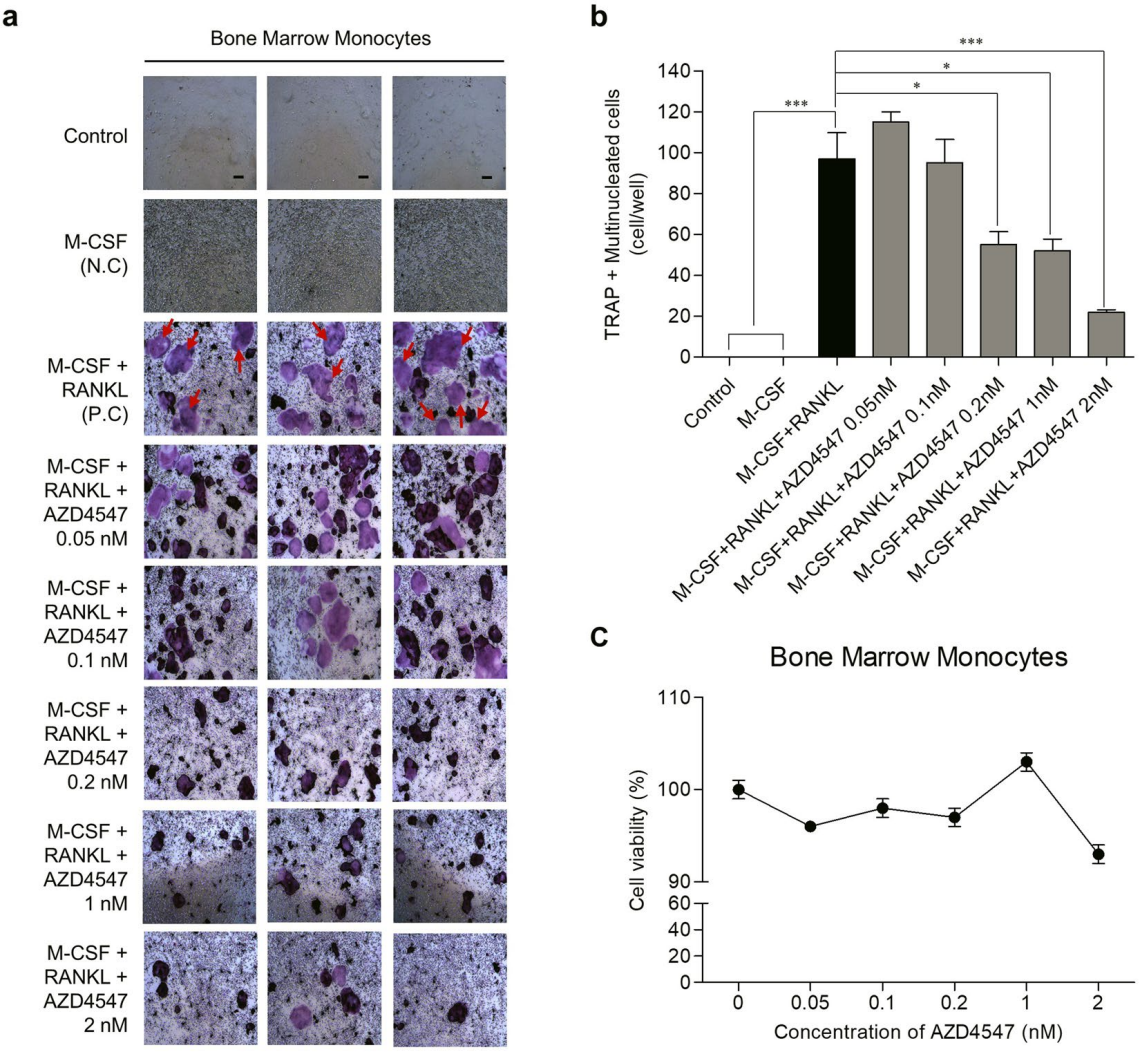
Effects of AZD4547 on osteoclastogenesis in mouse BMMs induced by RANKL and M-CSF. (**a**) Representative images of cell morphology in an osteoclastogenesis assay using mouse BMMs. BMMs were treated with M-CSF (30 ng/mL), RANKL (100 ng/mL), and AZD4547 (0.05–2 nM) for 1, 3, 5, and 7 days. Each point represents the mean of 3 independent determinations with the standard deviation. (**b**) The graph bars show mean number of TRAP + MNCs per well, and the error bars are the standard error. (**c**) Changes in cell survival following treatment with AZD4547 were determined in cytotoxicity assays. The percentage of viable BMMs after treatment with the indicated concentrations of AZD4547 for 48 h is shown. Each data point represents the mean of 6 independent determinations with the standard deviation. Red arrows, TRAP+ multinucleated cells; N.C, negative control; P.C, positive control; Scale bars, 100 µm. *p < 0.05, ***p < 0.001 in one-way ANOVA.

RANKL and M-CSF are known as key factors that promote osteoclastogenesis in the bone microenvironment, where cancer cells frequently metastasize. Therefore, we evaluated the effect of AZD4547 on osteoclast differentiation growth by conducting an osteoclastogenesis assay. Treatment with AZD4547 significantly decreased tartrate-resistant acid phosphatase-positive (TRAP+) multinucleated mouse bone marrow monocytes (BMMs) cells compared to the positive control (only RANKL and M-CSF stimulation) in a dose-dependent manner (Fig. 6a,b). Next, to demonstrate whether the effect of AZD4547 depends on inhibitory effect in TRAP+ mouse BMMs cells, a cytotoxicity assay was performed after AZD4547 treatment in mouse BMMs. As shown in Fig. 6c, the percentage of viable mouse BMMs cells was not decreased after AZD4547 treatment in a dose-dependent manner, showing no direct cytotoxicity of AZD4547 on mouse BMMs. These results demonstrate that AZD4547 has significant inhibitory effects on osteoclast differentiation growth induced by RANKL and M-CSF.

### AZD4547 modulates the bone microenvironment in FGFR non-amplified cancer-bearing mice

**Figure 7:**
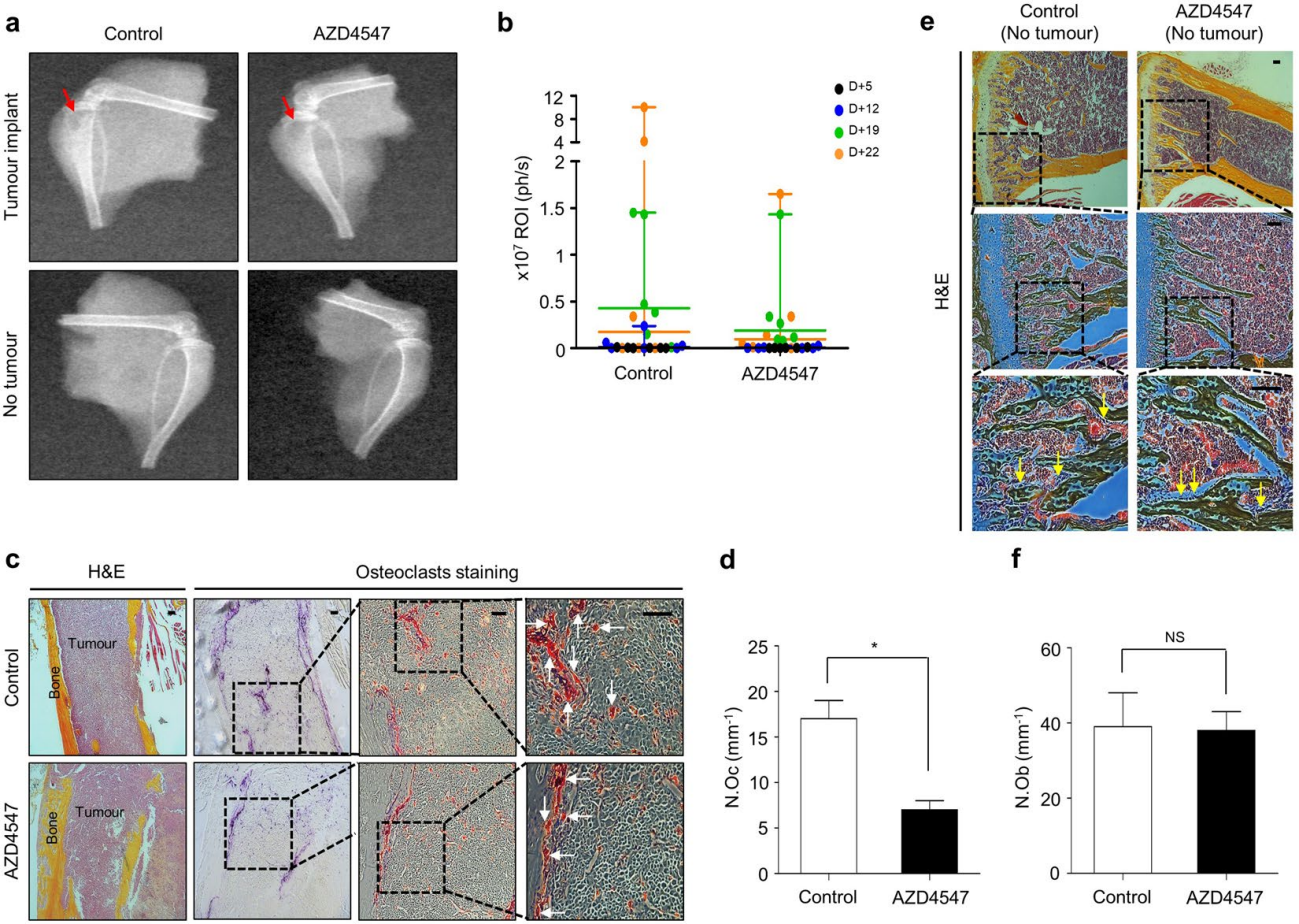
Interaction between FGFR non-amplified breast cancer cells and bone microenvironment was investigated with AZD4547. (**a**) AZD4547-insensitive MDA-MB-231-Luc cells were intratibially inoculated into mice (n = 6 per group), and each group of mice were treated daily with 0.5% carboxymethylcellulose sodium salt or AZD4547 (12.5 mg/kg) by oral gavage every 3 weeks. Representative radiographs of mouse hemi-tibiae and the mean tibiae from each group: control group and the AZD4547 group (upper/tumour implant and lower/no tumour). (**b**) Two experimental groups exhibited weekly tumour bioluminescence intensities *in vivo*. (**c**) Representative staining results from haematoxylin and eosin (H&E) and TRAP staining (osteoclast) in tumour-implanted tibiae tissues from each mouse groups. (**d**) The graph bars show the mean osteoclast numbers of whole tibiae, while error bars are the standard error. (**e**) Representative osteoblast staining results from H&E staining in no tumour-implanted tibiae tissues from each mouse groups. (**f**) The graph bars show mean numbers of osteoblasts (N.Ob) per square millimeter of bone surface (BS), while error bars are the standard error. Red arrow, osteolytic lesion area; white arrows, osteoclast; yellow arrows, osteoblast; Scale bars, 100 µm. *p < 0.05 compared to control in Student’s *t*-test.

To investigate how AZD4547 modulates the bone microenvironment through FGFR *in vivo*, we established a tumour-induced osteolysis model using the FGFR non-amplified breast cancer cell line MDA-MB-231 to evaluate the net effect of AZD4547 on the bone microenvironment, without the cancer cell killing effect. Radiographs of osteolytic lesions in the tibiae of mice injected with or without MDA-MB-231 cells are shown in Fig. 7a (red arrow). As shown in Fig. 7b, tumour growth in the tibiae was measured by weekly bioluminescence optical imaging (BLI) analysis. Tumour growth in the tibiae was not significantly different between the two experimental groups. Osteoclasts staining was performed by TRAP staining to examine osteoclasts in the tibiae tissues (Fig. 7c; white arrow). Although treatment with AZD4547 did not affect tumour size in the tibia, histopathological examination revealed that AZD4547 significantly decreased the number of osteoclasts compared to the control (17 in control group, 7 in AZD4547 group respectively, p < 0.05, Fig. 7d). Next, number of osteoblasts in non-tumour bearing mice was examined to exclude potential cytotoxic effect of AZD4547. Mean number of osteoblasts in tibiae after hematoxylin and eosin (H&E) staining (Fig. 7e, yellow arrow) was not significantly different between the two experimental groups (39 in control group, 38 in AZD4547 group respectively, p > 0.05, Fig. 7f). Taken together, these findings demonstrate that inhibition of the FGFR pathway can decrease osteoclastogenesis without inhibitory effect on osteoblasts in the bone microenvironment of metastasized cancer.

## Discussion

The current study was conducted to examine whether inhibition of FGFR by AZD4547 modulates the bone microenvironment, which is rich in cancer cell-derived cytokines that drive osteoclastogenesis. In oncology clinics, numerous new targets have been characterized to overcome pathologic bone resorption. The first-line treatment remains inhibition of osteoclasts using bisphosphonate or a monoclonal antibody targeting RANKL. In this study, we showed that AZD4547, a selective FGFR inhibitor, suppressed osteoclastogenesis by modulating osteoblasts *in vitro* and *in vivo*. AZD4547 inhibited the growth of osteoblastic cells and FGFR-amplified or -overexpressing breast cancer cells. FGF1 and the supernatant of an MDA-MB-134-VI cell culture induced the same pattern of RANKL/M-CSF/osteocalcin expression, which was suppressed by AZD4547. *In vitro*, osteoclastogenesis of BMMs stimulated by RANKL and M-CSF was significantly suppressed by AZD4547, which was supported by the results observed in the murine osteolytic bone model. These results indicate that inhibition of FGFR using AZD4547 significantly affects the modulation of the microenvironment in metastatic bone disease.

Several explanations have been proposed to describe how cancer cells interact with bone cells and the bone microenvironment^19^. The ‘vicious cycle hypothesis of bone metastasis’ first introduced by Mundy and colleagues in 1997, provides a reliable model explaining many aspects of the metastatic phenotype in bone^20,21^. The mineralized bone matrix is known to be rich in growth factors that are mobilized by osteoclastic resorption and become active in the local microenvironment. Release of these growth factors constitutes one of the key mechanisms in bone remodeling, allowing for physiological coupling between osteoblasts and osteoclasts^22^. Numerous soluble mediators released from cancer cells, such as interleukin-1 (IL-1), IL-6, IL-8, M-CSF, prostaglandin-E2, tumour necrosis factor-α and, most importantly, parathyroid hormone-related protein are known to induce osteoclastic differentiation^23^. Most of these factors contribute to osteolytic lesions via RANKL upregulation by osteoblasts and stromal cells. M-CSF serves as a cofactor in RANKL-stimulated differentiation of hematopoietic precursors into active osteoclasts^24^. In the current study, osteoblasts showed significantly higher RANKL and M-CSF expression following stimulation with the supernatant from MDA-MB-134-VI cells. However, this upregulation was inhibited by AZD4547. In a similar context, Aukes *et al*. demonstrated that the supernatant of MDA-MB-231-derived bone metastasis clones induced osteoclastogenesis^25^. Unlike our approach, they used the cell culture media as a source for tumour-derived FGF and evaluated the effect of FGFR TKI on osteoclastogenesis. These data suggest that FGFRs on osteoblasts are important mediators of the interaction between cancer and stromal cells in the bone microenvironment.

FGFR has been shown to be amplified in various cancer types, such as lung cancer^26^, oral squamous carcinoma^27,28^, pancreatic cancer^29^, and breast cancer^12,30,31^. Particularly, luminal B type breast cancers are commonly associated with FGFR amplification and bone metastasis, which are thought to be partly associated with FGFR amplification^12^. FGFs are potent regulators of bone metabolism. In bone tissues, FGF2 is produced by osteoblasts and functions as an autocrine/paracrine factor for various types of bone cells^32–34^. Recent data indicate that FGF2 and FGF8 stimulate osteoblasts and modulate bone formation in the presence of cancer cells, which in turn increase prostate cancer growth in bone^35,36^.

Many studies have investigated new drugs, particularly TKIs for targeting both the tumour and its interactions with the bone microenvironment. Most of these investigated drugs are multi-targeted TKIs, such as dovitinib (FGFR and VEGFR), imatinib (PDGF, c-Abl, c-KIT, and c-Fms receptor), and cabozantinib (MET and VEGFR2)^15,37,38^. Although previous studies demonstrated that multi-TKIs have modulating effects on the bone microenvironment, the effects may result from the combined anti-tumour activity and inhibitory effects on osteoblastic cells. Thus, the contribution of each receptor tyrosine kinase to regulating of bone remodelling is difficult to determine. In the current study, we used MDA-MB-231 cells for *in vivo* analysis to identify the effect of AZD4547 in the bone microenvironment. AZD4547 significantly decreased osteoclastogenesis in the murine model, without any direct anti-tumour effects on metastatic bone cancer. Therefore, our data suggest that the FGF/FGFR pathway is a potential target for metastatic bone disease.

Anti-resorptive agents, such as bisphosphonates and RANKL-targeting denosumab, are typically part of the treatment plan for cancer patients with bone metastasis. Because of the potent inhibitory effects of these agents on osteoclasts, there are important concerns regarding the development of osteonecrosis of the jaw because bone healing is impaired. There are also concerns regarding the potential harmful effects of FGFR blockade on normal bone remodelling, as AZD4547 inhibits the secretion of RANKL/M-CSF by osteoblasts and differentiation of osteoclasts in the absence of tumour cells. However, AZD4547 showed little cytotoxicity towards MC3T3-E1 cells (osteoblasts) when used at lower doses in the MTT assay. Additionally, peritumoral stromal cells may be more sensitive to AZD4547 than normal stromal cells because peritumoral stromal cells have pre-existing FGFR activation through the activity of various cancer cell-secreted cytokines as a paracrine signal. Previous clinical studies targeting FGFR found no significant increase in serious side effects on the musculoskeletal system^12,13^. Thus, the effects of AZD4547 on normal bone remodelling may be minimal.

In conclusion, we showed that the selective FGFR inhibitor AZD4547 suppresses osteoclastogenesis via a mechanism of decreased RANKL/M-CSF expression in metastatic bone microenvironment.

## Materials and Methods

### Cell culture and reagents

The murine pre-osteoblastic cell line (MC3T3-E1 Subclone 4), human osteosarcoma cell line (MG-63), and human breast cancer cell lines (MDA-MB-134-VI, MDA-MB-231, and MCF-7) were purchased from American Type Culture Collection (Manassas, VA, USA). The MDA-MB-231-Luc cell line was kindly provided by Associate Professor Serk In Park (Laboratory of Biochemistry and Molecular Biology, Seoul, Korea). MC3T3-E1 cells were maintained in Minimum Essential Medium Alpha Modification (α-MEM) (LM008-01, Welgene Inc., Daegu, Korea) supplemented with 10% foetal bovine serum, 1% penicillin-streptomycin and 2 mM L-glutamine. MG-63 cells were maintained in DMEM (Welgene Inc.; LM001-05) supplemented with 10% foetal bovine serum, 1% penicillin-streptomycin and 2 mM L-glutamine. MDA-MB-134-VI cells were maintained in Leibovitz’s L-15 medium (11415-064, Invitrogen, Carlsbad, CA, USA) supplemented with 20% foetal bovine serum, 1% penicillin-streptomycin and 2 mM L-glutamine. MDA-MB-231 cells were maintained in RPMI-1640 medium (SH30027.01, Thermo Fisher Scientific Inc., Waltham, MA, USA) supplemented with 10% foetal bovine serum, 100 U/mL penicillin, 100 mg/mL streptomycin and 2 mM L-glutamine. MCF-7 cells were maintained in RPMI-1640 medium supplemented with 4 mg/mL insulin human recombinant zinc solution (Invitrogen; 12585-014), 10% foetal bovine serum, 100 U/mL penicillin, 100 mg/mL streptomycin and 2 mM L-glutamine. MDA-MB-134-VI cells, were incubated in a humidified 37 °C incubator. All cultured cells, except for MDA-MB-134-VI cells were incubated in a humidified 37 °C incubator with 5% CO_2_. AZD4547 and BGJ398 were obtained from Selleckchem; S2801, S2183 (Houston, TX, USA). Recombinant mouse FGF-1 and human FGF-1 were obtained from R&D Systems; 4686-FA-025 (Minneapolis, MN, USA) and Peprotech; 100–17 A (Rocky Hill, NJ, USA). RANKL and M-CSF were obtained from Peprotech; 315-11 and 315-02.

### Cell viability assay

Cells were seeded at a density of 2 × 10^5^ cells/well into 6-well plates. After 24 h, the cells were treated with various concentrations of AZD4547. Cell viability was quantitatively analysed using the 3-[4,5-dimethylthiazol-2-yl]-2,5-diphenyltetrazolium (MTT) assay. Briefly, 500 μg/mL of MTT solution (5 mg/mL in 1 × PBS; M2128, Sigma Aldrich, St. Louis, MO, USA) was added to each well after treatment with AZD4547 and further incubated for 4 h at 37 °C. The medium was removed and 1 mL of dimethyl sulfoxide (DMSO; D8418, Sigma Aldrich) was added to each well. The absorbance of the converted MTT dye was measured at 540 nm using an iMARK microplate reader (Bio-Rad Laboratories, Hercules, CA, USA). Cell viability was expressed as the relative percentage compared to untreated cells; experimental error was also calculated. All experiments were repeated independently 3 times. The percent of cell survival was calculated using GraphPad Prism software version 5.01 (GraphPad Inc., La Jolla, CA, USA).

### Quantitative reverse transcription-polymerase chain reaction (qRT-PCR)

Total RNA was isolated from cells using TRIzol reagent (Invitrogen; 10296-010), according to the manufacturer’s protocol. The RNA was reverse transcribed into cDNA, and real-time PCR analysis was performed using the CFX96 Real-Time System (Bio-Rad) with iQ^TM^ SYBR Green Supermix (Bio-Rad; 170–8882AP). The PCR protocol was as follows: 1 cycle of 95 °C for 5 min; 40 cycles of 95 °C for 30 s, 65 °C for 30 s, and 72 °C for 1 min; 1 cycle of 95 °C for 1 min, 55 °C for 1 min; 1 cycle of 60 °C for 10 s. Quantitative PCR was performed using specific primers to amplify Tnfsf11 (sense, 5′-TCGCTCTGTTCCTGTACT-3′, and antisense, 5′-AGTGCTTCTGTGTCTTCG-3′), Csf1 (sense, 5′-CCCATATTGCGACACCGAA-3′, and antisense, 5′-AAGCAGTAACTGAGCAACGGG-3′). All experiments were repeated independently 3 times. The relative expression was calculated using Bio-Rad CFX manager software version 3.0 (Bio-Rad).

### M-CSF and OPG ELISA

The levels of M-CSF and OPG in the cell culture (MC3T3-E1 and MG-63) supernatants were analyzed with the Mouse and Human M-CSF Quantikine^TM^ ELISA kit (R&D Systems; MMC00 and DMC00B), and OPG Quantikine^TM^ ELISA kit (R&D Systems; MOP00 and DY805) in accordance with the manufacturer’s protocols. Briefly, 50 µL of assay diluents were added to wells pre-coated with a monoclonal antibody specific for M-CSF and OPG. Subsequently, 50 µL of control standard or a 4-fold dilution of cell culture supernatants was added to each well and incubated for 2 h at room temperature. The optical density of each well at 450 nm was determined using an iMARK^TM^ microplate reader (Bio-Rad). All cell culture supernatants were measured 3 times, and the mean measurements were used for analysis.

### Western blot analysis

Cells were harvested in PRO-PREP^TM^ Protein Extraction Solution (17081, Intron, Gyeonggi, Korea) supplemented with Xpert phosphatase inhibitor cocktail solution (P3200, genDEPOT, Katy, TX, USA) and incubated on ice for 20 min. Protein concentrations were measured using Bio-Rad protein assay kits. Proteins (50 µg) were separated by electrophoresis on 8% or 12% SDS-polyacrylamide gels and transferred to polyvinylidene fluoride membranes (10600023, GE Healthcare, Little Chalfont, UK). The membranes were blocked with 5% non-fat milk in Tris-buffered saline containing 0.1% Tween 20 (8571-1405, Dae Jung, Gyeonggi, Korea) for 1 h at room temperature and then incubated with the following primary antibodies overnight at 4 °C: RANKL (sc-9073, Santa Cruz Biotechnology, Dallas, TX, USA), FGFR1 (9740, Cell Signaling Technology, Danvers, MA, USA), FGFR2 and phosphorylated FGFR1 (ab109372 and ab173305, Abcam, Cambridge, UK). After washing 3 times in Tris-buffered saline-Tween, the membrane was incubated for 1 h at room temperature with anti-mouse or anti-rabbit horseradish peroxidase-conjugated secondary antibodies (Bio-Rad; 170–6516 and Santa Cruz Biotechnology; sc-2004). Antibody-protein complexes were visualized using an ECL system (GE Healthcare; RPN2106) and the images were developed on X-ray film (CP-BU, Agfa Healthcare, Mortsel, Belgium). The original gels are shown in the Supplementary File. Band density was analyzed with QuantityOne^TM^ image analysis software (Bio-Rad).

### Osteoclastogenesis assay

Primary mouse bone marrow monocytes (BMMs) were isolated from 2 femurs and 2 tibiae of 6–8-week-old C57BL/6 mice. Mouse BMMs were flushed into a 15-mL tube with 8 mL of serum-free α-MEM containing 2 mM EDTA and then separated with lymphocyte separation medium (50494×, MP Biomedicals, Illkirch, France). BMMs were cultured in α-MEM containing 10% heat inactivated foetal bovine serum. For the assay, BMMs were seeded at 2 × 10^5^ cells/well into 48-well plates with 0.5 mL of medium per well. The BMMs were then stimulated with M-CSF (30 ng/mL) for the negative control group, M-CSF and RANKL (100 ng/mL) for the positive control group, and M-CSF, RANKL, and AZD4547 (0.05, 0.1, 0.2, 1 and 2 nM) for the experimental group. Cells were cultured in a humidified 37 °C incubator with 5% CO_2_. After 1, 3, 5, and 7 days of culture, the medium was changed and replenished with complete medium with M-CSF with the experimental treatments (RANKL with or without AZD4547). After 9 days of culture, the cells were fixed with 10% formalin and stained to assay tartrate-resistant acid phosphatase (TRAP) activity using a TRAP staining kit (KT-008, KAMIYA biomedical company, Seattle, WA, USA). We photographed and counted the TRAP-positive (TRAP+) multinucleated cells (TRAP + MNCs) in each well. All experiments were repeated independently 3 times. All experimental procedures involving mice were performed with the guidance and protocols approval by the Institutional Animal Care and Use Committee of Korea University (Approval number; KUIACUC-2016-37). All method were carried out in accordance with the relevant guidelines and regulations.

### Cytotoxicity assay

Primary mouse BMMs were isolated from 2 femurs and 2 tibiae of 6–8-week-old C57BL/6 mice. Mouse BMMs were flushed into a 15-mL tube with 8 mL of serum-free α-MEM containing 2 mM EDTA and then separated with lymphocyte separation medium (50494×, MP Biomedicals, Illkirch, France). BMMs were cultured in α-MEM containing 10% heat inactivated foetal bovine serum. For the assay, BMMs were seeded at 2 × 10^5^ cells/well into 48-well plates with 0.5 mL of medium per well. The BMMs were then stimulated with M-CSF (30 ng/mL) for the negative control group, M-CSF and AZD4547 (0.05, 0.1, 0.2, 1 and 2 nM) for the experimental group. Cells were cultured in a humidified 37 °C incubator with 5% CO_2_. After 48 h, 500 μg/mL of MTT solution (Sigma Aldrich) was added to each well and further incubated for 4 h at 37 °C. The medium was removed and 300 μL/well of DMSO (Sigma Aldrich) was added to each well. The absorbance of the converted MTT dye was measured at 540 nm using an iMARK microplate reader (Bio-Rad Laboratories). Cell viability was expressed as the relative percentage compared to untreated cells; experimental error was also calculated. All experiments were repeated independently 3 times. The percent of cell survival was calculated using GraphPad Prism software version 5.01 (GraphPad Inc.).

### Animal study

Six to eight-week-old female BALB/c nu/nu mice (10 per group) were purchased from Orient Bio, Inc. (Gyeonggi, Korea). MDA-MB-231-Luc cells (2 × 10^5^) in 20 µL of PBS were injected into the right tibiae of mice in both groups under appropriate anaesthetics and analgesics. One week after injection, the mice were treated daily by oral gavage with AZD4547 (12.5 mg/kg; experimental group, n = 6) in 0.5% carboxymethylcellulose sodium salt (CMC-Na, Sigma Aldrich; C5678) or with 0.5% CMC-Na (control group, n = 6) every 3 weeks. The concentration of AZD4547 were selected in accordance with the manufacturer’s instructions. All mice were sacrificed on day 28, and X-ray and BLI images of the tibiae were obtained to evaluate the tumour burden with a NightOWL II LB 983 *in vivo* imaging system (BERTHOLD Technologies, Bad Wildband, Germany). The areas of osteolytic or sclerotic lesions were quantified by using Indig software (BERTHOLD Technologies). Tibiae tissues were collected for histological analysis. All experimental procedures involving mice were performed with the guidance protocols approval by the Institutional Animal Care and Use Committee of Korea University (Approval number; KUIACUC-2016-37). All method were carried out in accordance with the relevant guidelines and regulations.

### Hematoxylin and Eosin (H&E) and osteoclasts staining

Tumour-bearing or contralateral tibiae were fixed with 2% paraformaldehyde (Sigma) for 4 days, and then decalcified with 20% EDTA (Sigma Aldrich; E5134) for 14 days (EDTA was changed every other day). Next, the bones were stored in 70% ethanol and embedded in paraffin. The section (8 µm) were stained with haematoxylin and eosin (H&E) using standard techniques for microscopic examination. Paraffin-embedded tibiae tissue slices were stained using a TRAP staining kit (Osteoclasts staining; KAMIYA Biomedical Company). The extent of new bone microenvironments was determined as the areas in the middle sections, from levels 2 to 7, stained with H&E and TRAP. The stained tibiae images were captured using a Z1 Carl Zeiss microscope equipped with an Axio scan (Zeiss, Gottingen, Germany). Optical density values were determined by Axioscan Image processing software (Zeiss).

### Statistics analysis

All statistical analyses were performed using GraphPad Prism software (version 5.01). Differences in osteoclast number between the two experimental groups were evaluated by Students *t*-test. Otherwise, differences between multiple experimental groups were examined by one-way analysis of variance (ANOVA) followed by the Tukey test. The two-way ANOVA test was used to evaluate differences in cell survival using the MTT assay results. In all cases, a *p* value of <0.05 was considered significant.

## Supplementary information

Supplementary data infomation
