# Supplementary material for: A Selective FGFR inhibitor AZD4547 suppresses RANKL/M-CSF/OPG-dependent ostoclastogenesis and breast cancer growth in the metastatic bone microenvironment
Source: Sci Rep. 2019 Jun 19;9:8726. doi: 10.1038/s41598-019-45278-w (PMC6584658; doi:10.1038/s41598-019-45278-w)
Supplement: Supplementary file 1 — Supplementary data infomation [file 41598_2019_45278_MOESM1_ESM.pdf]

A selective FGFR inhibitor AZD4547 suppresses RANKL/M-CSF/OPG-dependent osteoclastogenesis and breast cancer growth in the metastatic bone microenvironment

Jinho Kang[1,2], Yoon Ji Choi[2], Bo Yeon Seo[1,3], Ukhyun Jo[2], Serk In Park[1,3,4], Yeul Hong Kim[1,2], Kyong Hwa Park[1,2,*]

[1]The BK21 Plus Program, Korea University College of Medicine, Seongbuk-Gu, Seoul, Republic of Korea.

[2]Division of Oncology/Hematology, Department of Internal Medicine, Korea University College of Medicine, Seongbuk-Gu, Seoul, Republic of Korea.

[3]Department of Biochemistry and Molecular Biology, Korea University College of Medicine, Seongbuk-Gu, Seoul, Republic of Korea. [4]Department of Medicine, Vanderbilt University School of Medicine, Nashville, TN, USA.

*Corresponding author: Kyong Hwa Park MD, PhD

E-mail: khpark@korea.ac.kr

Original gels data (Figure 1)

a

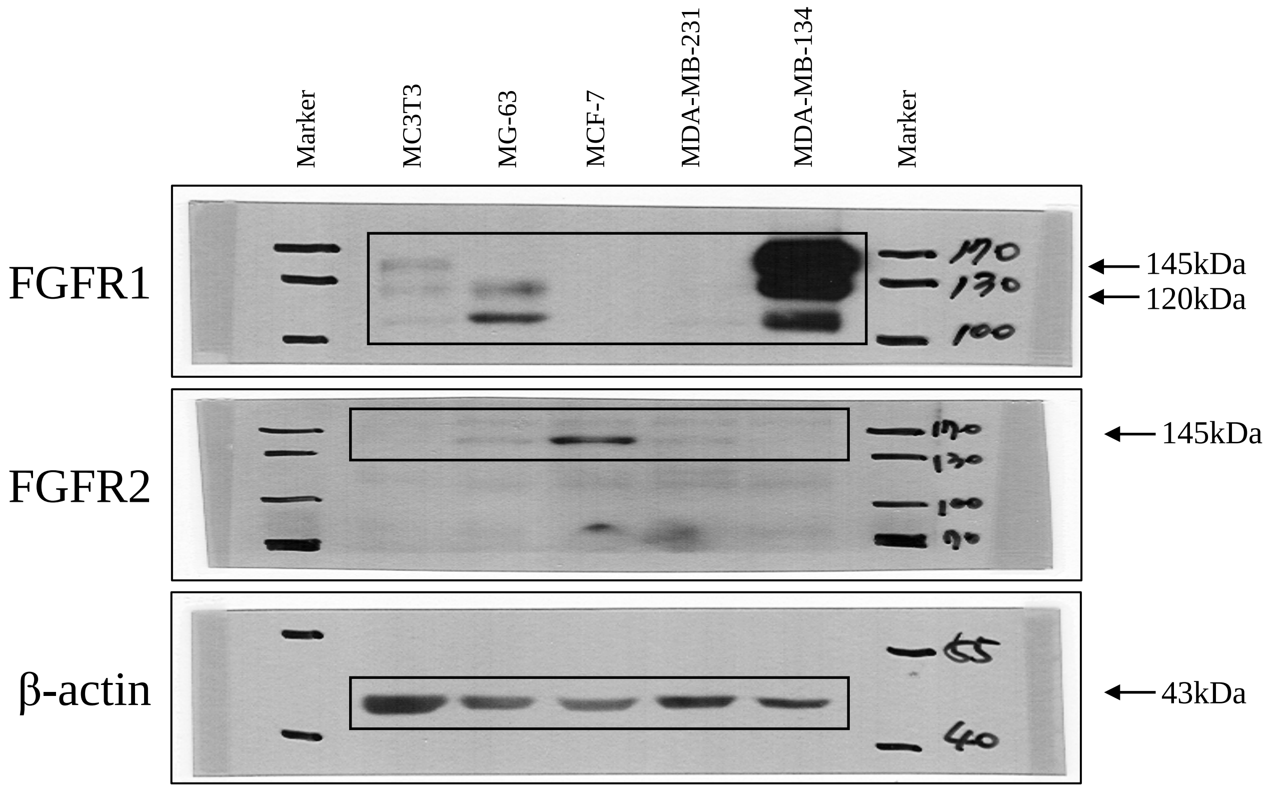

Original gels data (Figure 2)

a MC3T3-E1

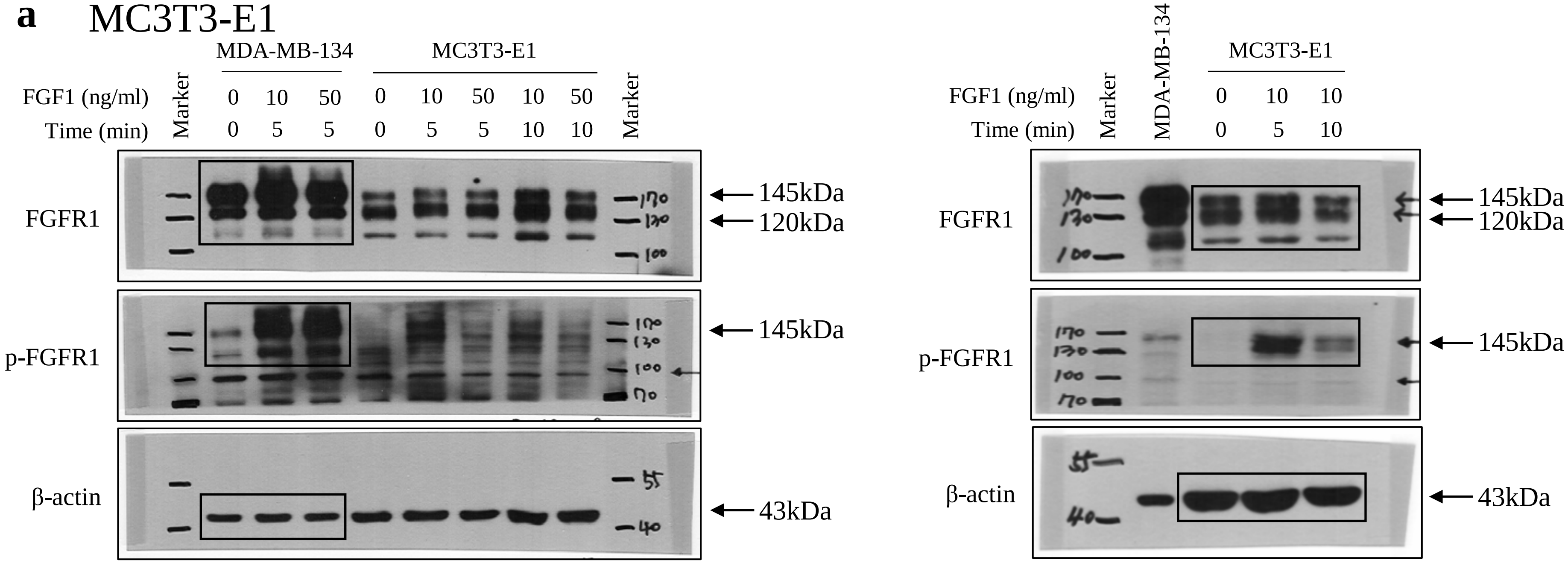

MG-63

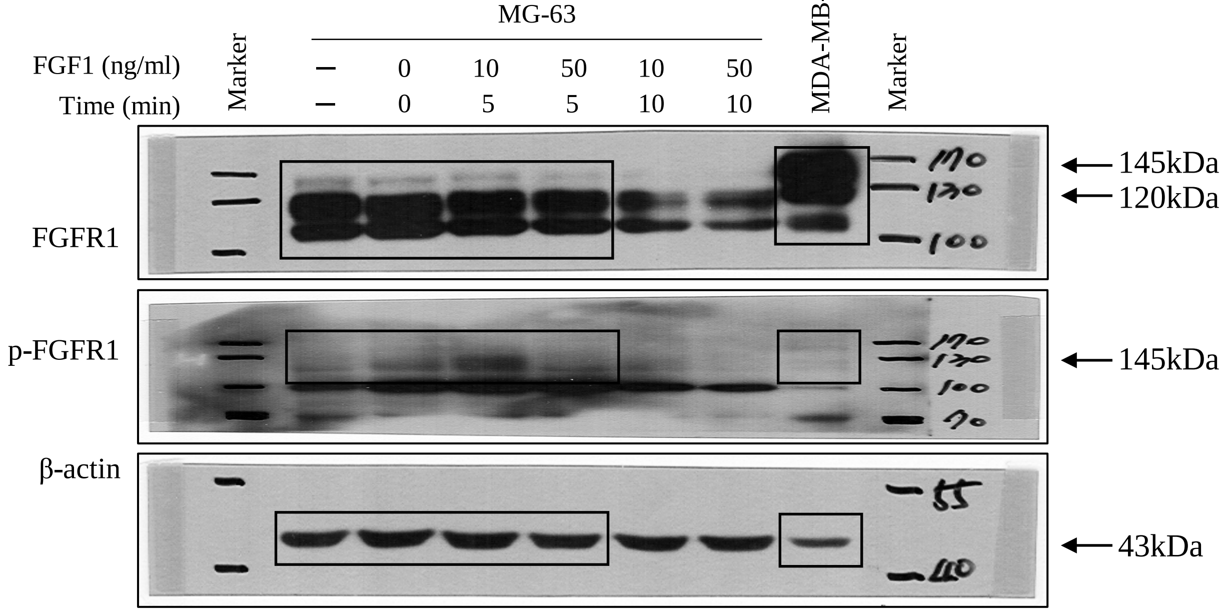

Original gels data (Figure 2)

b MC3T3-E1

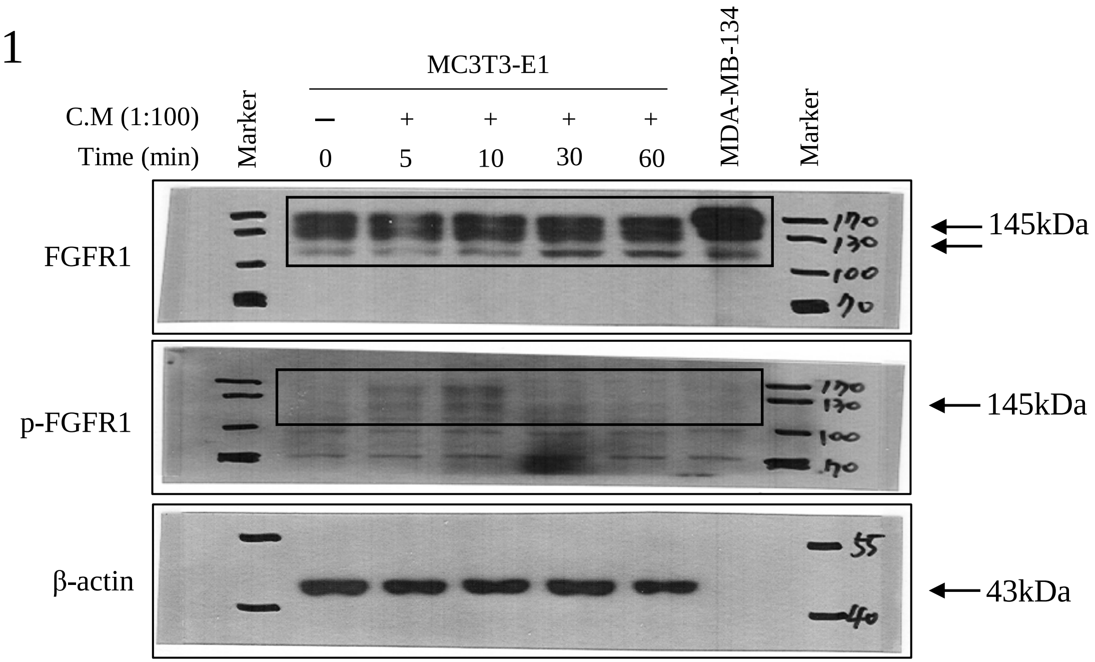

MG-63

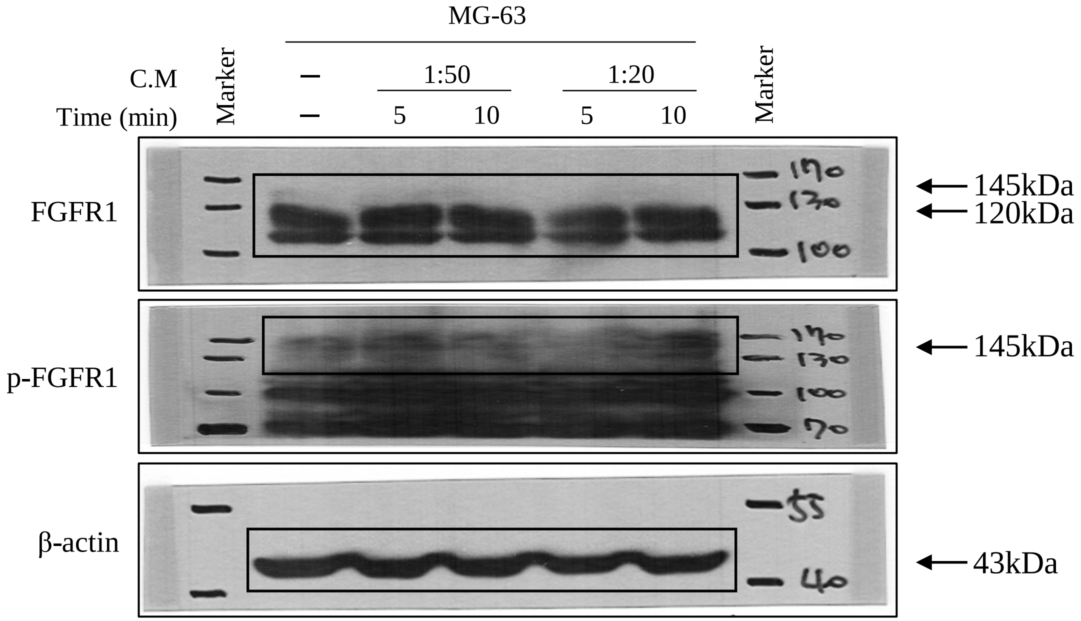

Original gels data (Figure 5)

a

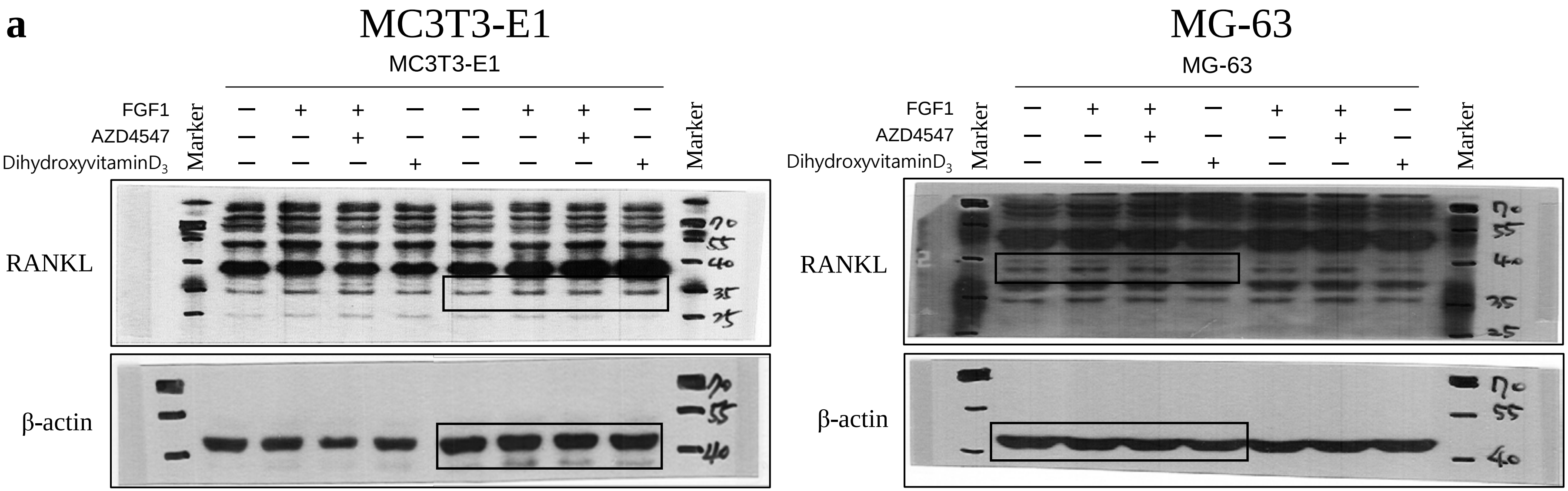

b

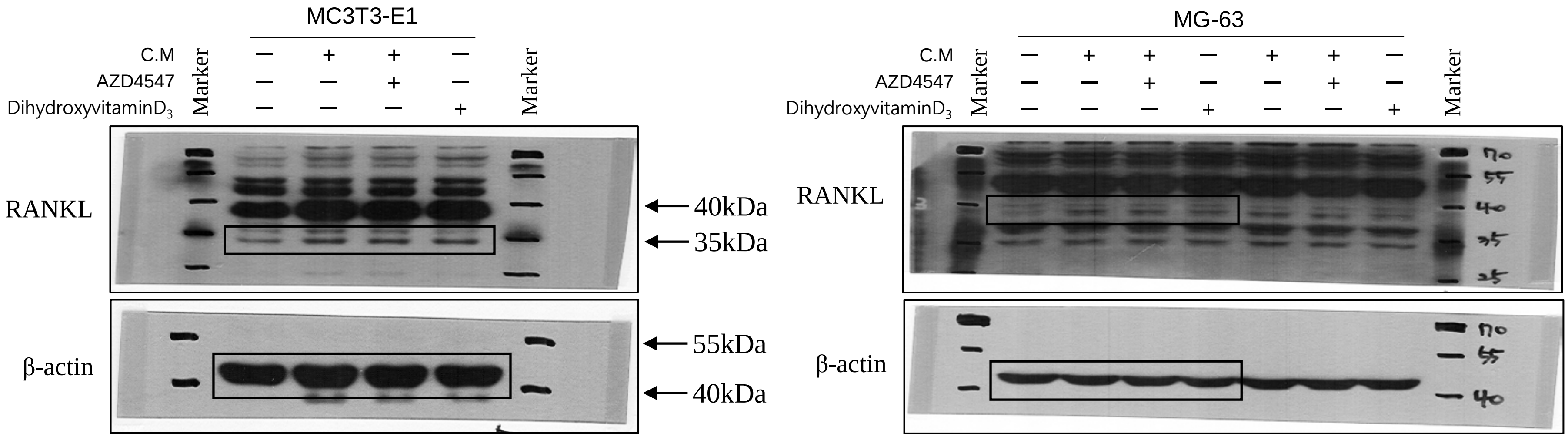

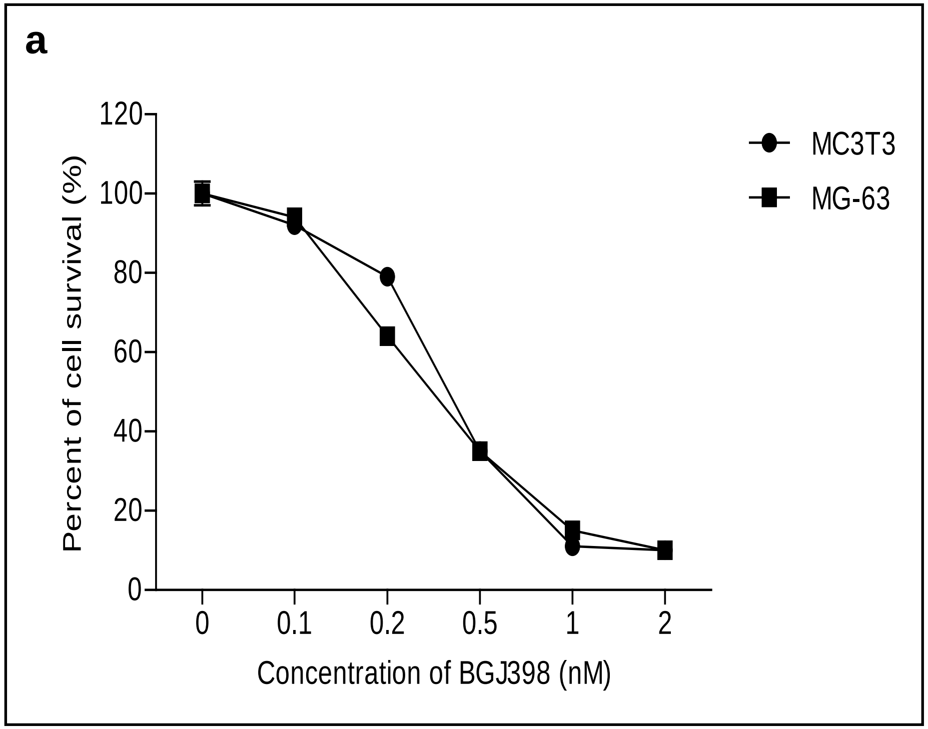

Supplementary Figure S1. Evaluation of BGJ398 sensitivity of pre-osteoblast cells.

(a) Changes in cell survival following treatment with BGJ398 were determined in MTT assays. The percentage of viable cells after treatment with the indicated concentrations of BGJ398 for 24 h is shown. Each data point represents the mean of 6 independent determinations with the standard deviation.

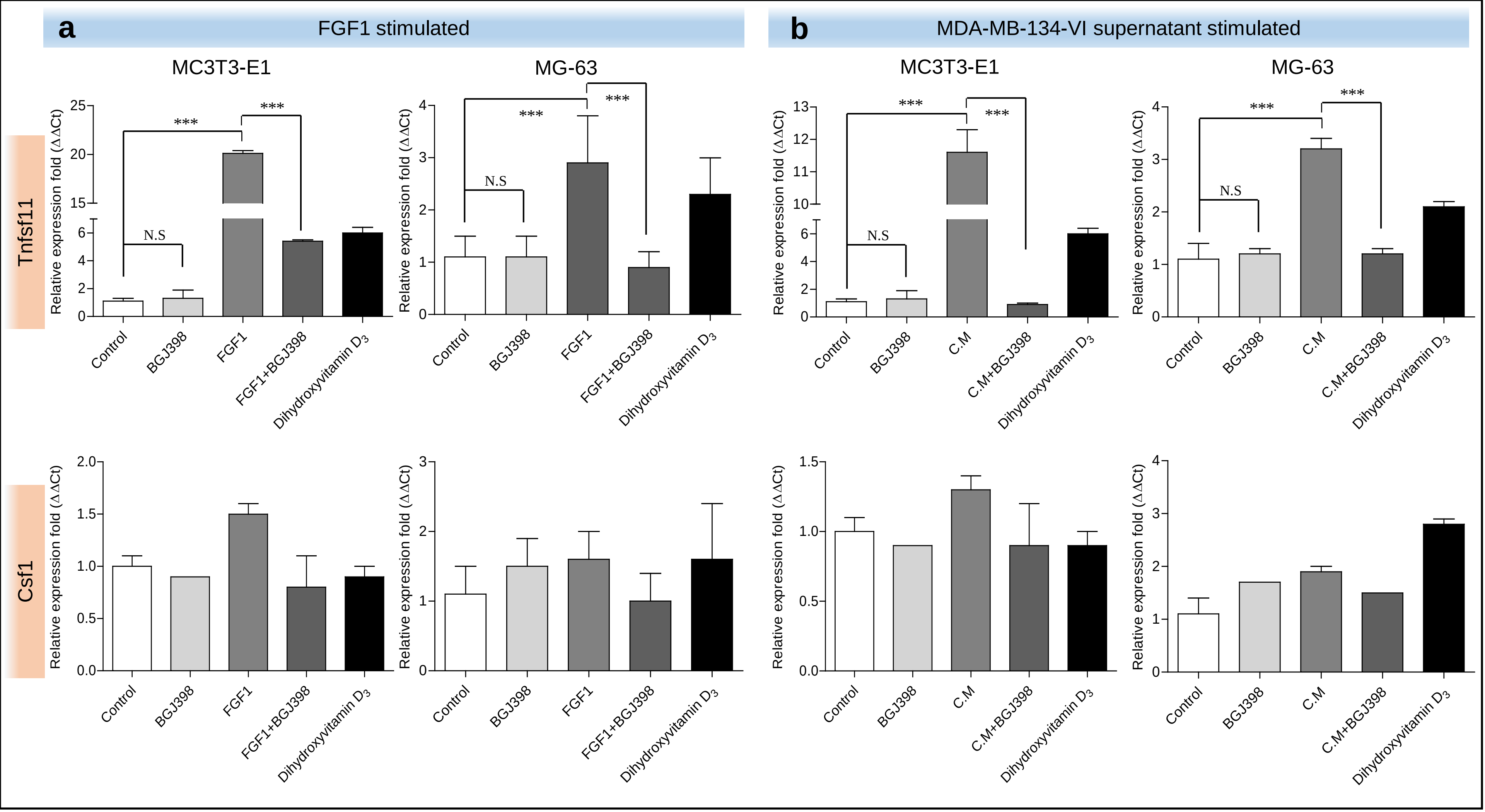

Supplementary Figure S2. Effects of BGJ398 on expression of RANKL, M-CSF following FGFR activation induced by FGF-1 and MDA-MB-134-VI supernatant.

Effects of AZD4547 on FGF1-stimulated (a) and MDA-MB-134-VI supernatant-stimulated (b) expression of osteoblast-related genes (Tnfsf11 and Csf1) in pre-osteoblast cells were examined by quantitative RT-PCR. Cells were stimulated with FGF1 (10 ng/mL) or MDA-MB-134-VI supernatant (diluted 1:20), and then treated with BGJ398 (0.2 nM) for 2 h before total RNA lysates were prepared. Each data point represents the mean value acquired from 3 experiments with the standard error. Dihydroxyvitamin D_3 is mean as positive control of osteoblasts. C.M, MDA-MB-134-VI supernatant. *** $P < 0.05$, in one-way ANOVA.

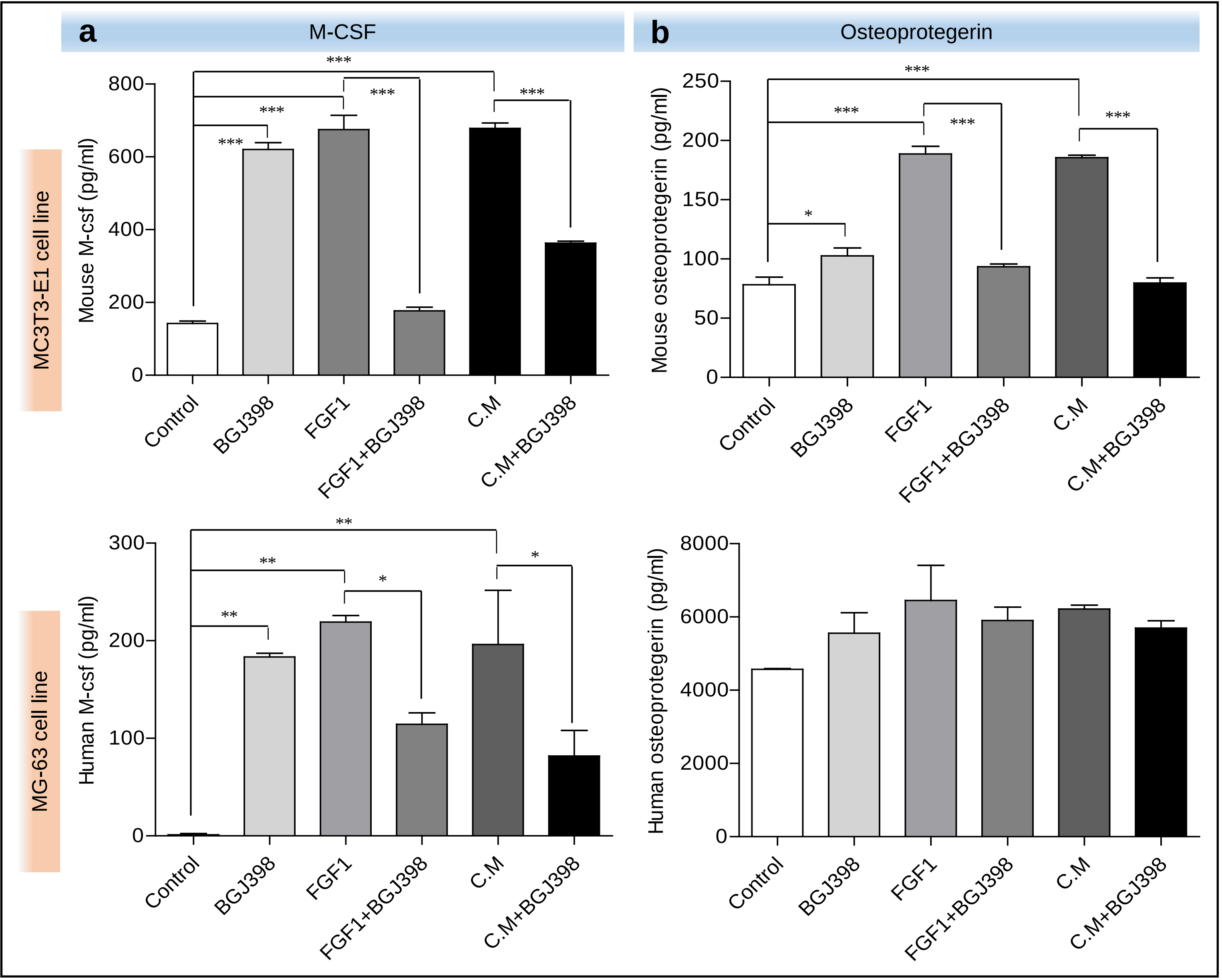

Supplementary Figure S3. M-CSF and OPG expression levels induced by FGF-1 or MDA-MB-134-VI supernatants are reduced by BGJ398.

The effects of AZD4547 on FGF1 (10 ng/mL)- or MDA-MB-134-VI supernatants (diluted 1:20)-induced M-CSF (a) and OPG (b) expression was examined using ELISA after treatment with BGJ398 (0.2 nM) in MC3T3 and MG-63 cells. The optical density of each well at 450 nm was determined using an iMARK^{TM} microplate reader. Each point represents the mean of 3 independent determinations with the standard deviation. C.M, MDA-MB-134-VI supernatant. * $P < 0.05$, in one-way ANOVA.
